# Digital breast tomosynthesis in mammographic screening: false negative cancer cases in the To-Be 1 trial

**DOI:** 10.1186/s13244-023-01604-5

**Published:** 2024-02-08

**Authors:** Nataliia Moshina, Axel Gräwingholt, Kristina Lång, Ritse Mann, Tone Hovda, Solveig Roth Hoff, Per Skaane, Christoph I. Lee, Hildegunn S. Aase, Aslak B. Aslaksen, Solveig Hofvind

**Affiliations:** 1 Section for Breast Cancer Screening, Cancer Registry of Norway, Oslo, Norway; 2 Mammographiescreening-Zentrum Paderborn, Breast Cancer Screening, Paderborn, NRW Germany; 3 Department of Translational Medicine, Lund University, Lund, Sweden; 4 Department of Medical Imaging, Radboud University Medical Center, Nijmegen, the Netherlands; 5 Department of Radiology, the Netherlands Cancer Institute, Amsterdam, the Netherlands; 6 Department of Radiology, Vestre Viken Hospital Trust, Drammen, Norway; 7 Department of Radiology, Ålesund Hospital, Møre Og Romsdal Hospital Trust, Ålesund, Norway; 8 Department of Circulation and Medical Imaging, Faculty of Medicine and Health Sciences, NTNU, Trondheim, Norway; 9 Department of Radiology, Oslo University Hospital, University of Oslo, Oslo, Norway; 10 Department of Radiology, University of Washington School of Medicine, Seattle, WA USA; 11 Department of Health Systems and Population Health, University of Washington School of Public Health, Seattle, WA USA; 12 Department of Radiology, Haukeland University Hospital, Bergen, Norway; 13 Department of Global Public Health and Primary Care, Faculty of Medicine, University of Bergen, Bergen, Norway; 14 Department of Health and Care Sciences, Faculty of Health Sciences, UiT, The Arctic University of Norway, Tromsø, Norway

**Keywords:** Mammographic screening, Breast cancer, Digital breast tomosynthesis, Interval cancer, Screen-detected cancer

## Abstract

**Objectives:**

The randomized controlled trial comparing digital breast tomosynthesis and synthetic 2D mammograms (DBT + SM) versus digital mammography (DM) (the To-Be 1 trial), 2016–2017, did not result in higher cancer detection for DBT + SM. We aimed to determine if negative cases prior to interval and consecutive screen-detected cancers from DBT + SM were due to interpretive error.

**Methods:**

Five external breast radiologists performed the individual blinded review of 239 screening examinations (90 true negative, 39 false positive, 19 prior to interval cancer, and 91 prior to consecutive screen-detected cancer) and the informed consensus review of examinations prior to interval and screen-detected cancers (*n* = 110). The reviewers marked suspicious findings with a score of 1–5 (probability of malignancy). A case was false negative if ≥ 2 radiologists assigned the cancer site with a score of ≥ 2 in the blinded review and if the case was assigned as false negative by a consensus in the informed review.

**Results:**

In the informed review, 5.3% of examinations prior to interval cancer and 18.7% prior to consecutive round screen-detected cancer were considered false negative. In the blinded review, 10.6% of examinations prior to interval cancer and 42.9% prior to consecutive round screen-detected cancer were scored ≥ 2. A score of ≥ 2 was assigned to 47.8% of negative and 89.7% of false positive examinations.

**Conclusions:**

The false negative rates were consistent with those of prior DM reviews, indicating that the lack of higher cancer detection for DBT + SM versus DM in the To-Be 1 trial is complex and not due to interpretive error alone.

**Critical relevance statement:**

The randomized controlled trial on digital breast tomosynthesis and synthetic 2D mammograms (DBT) and digital mammography (DM), 2016–2017, showed no difference in cancer detection for the two techniques. The rates of false negative screening examinations prior to interval and consecutive screen-detected cancer for DBT were consistent with the rates in prior DM reviews, indicating that the non-superior DBT performance in the trial might not be due to interpretive error alone.

**Key points:**

• Screening with digital breast tomosynthesis (DBT) did not result in a higher breast cancer detection rate compared to screening with digital mammography (DM) in the To-Be 1 trial.

• The false negative rates for examinations prior to interval and consecutive screen-detected cancer for DBT were determined in the trial to test if the lack of differences was due to interpretive error.

• The false negative rates were consistent with those of prior DM reviews, indicating that the lack of higher cancer detection for DBT versus DM was complex and not due to interpretive error alone.

**Graphical Abstract:**

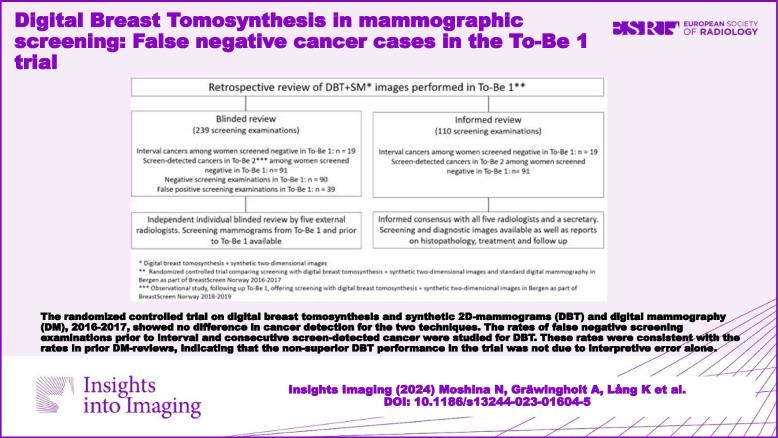

**Supplementary Information:**

The online version contains supplementary material available at 10.1186/s13244-023-01604-5.

## Introduction

Studies have shown that breast cancer screening with digital breast tomosynthesis (DBT) in combination with standard digital mammograms (DM) or synthetic 2D mammograms (SM) is associated with higher rates of screen-detected cancer compared to standard DM [1–4]. The effect of DBT on interval cancer rates is still unclear due to small number of cases included in studies [5]. Only one study has reported a reduction in interval cancers among those screened with DBT versus DM [6]. In 2021, a meta-analysis of pooled data from prospective European trials and observational US studies showed that screening with DBT resulted in an increase in screen-detected cancers in Europe, while the recall rates decreased to a larger extent in the USA [5].

The Tomosynthesis trial in Bergen (To-Be 1) was a randomized controlled trial (RCT) comparing DBT + SM and DM in BreastScreen Norway, 2016–2017 [7]. The trial did not reveal a statistically significant higher cancer detection rate for DBT + SM [7]. In the follow-up study, To-Be 2, a prospective cohort study, all women were offered screening with DBT + SM. A higher cancer detection rate was observed in To-Be 2 in contrast to To-Be 1 [8]. It is unclear why there was no difference in cancer detection for DBT + SM compared to DM in To-Be 1 [7, 9].

Retrospective blinded reviews have shown varying rates of false negatives in mammography screening studies due to different study designs and definitions of false-negative examinations [10–17]. Previous informed consensus-based reviews have classified 13–33% of the screen-detected and interval cancers from mammography studies as false negative [14–16, 18]. To shed light on possible reasons for nonsignificant differences between DBT + SM and DM screening in To-Be 1, we invited five expert breast radiologists not involved in the To-Be trial to perform a retrospective review of DBT + SM screening examinations resulting in interval cancers detected in To-Be 1 and screen-detected cancers in To-Be 2. The objective was to determine whether interval cancers in To-Be 1 and screen-detected cancers in To-Be 2 were due to interpretive error in the To-Be 1 trial.

## Material and methods

The To-Be trials were approved by the Regional Committee for Medical and Health Research Ethics in Norway (no. 2015/424) and registered at ClinicalTrials.gov (NCT02835625 and NCT03669926). All women participating in the To-Be trials signed an informed consent.

The two prospective trials were performed in Bergen, as a part of BreastScreen Norway, an organized population-based screening program, administered by the Cancer Registry of Norway [7, 19].

The trials are described in detail elsewhere [7, 8]. Briefly, To-Be 1 was a randomized controlled trial (RCT) comparing screening outcomes of DBT + SM, with standard DM. To-Be 1 recruited women in 2016–2017 and randomly assigned them to screening with either two view DBT + SM or DM [7]. In the following 2 years, 2018–2019, To-Be 2 was performed where all enrolled women were screened with DBT + SM [8]. All screening examinations were independently read by two radiologists using an interpretation score ranging from 1 to 5 for each breast. A score of 1 indicated a negative result, 2 probably benign, 3 intermediate suspicion, 4 probably malignant, and 5 high suspicions of malignancy. All cases with a score of 2 or higher by one or both radiologists were discussed at consensus to determine if a woman should be recalled. The examinations were performed with GE Healthcare units, SenoClaire in To-Be 1, and with Senographe Pristina in To-Be 2.

### Blinded review

**Fig. 1:**
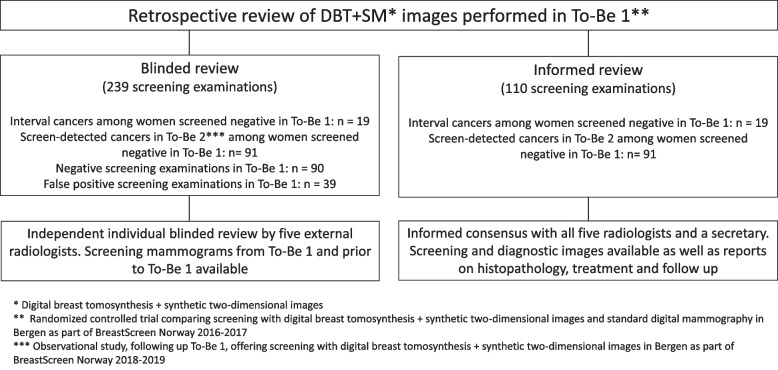
Study design for the blinded individual and informed consensus-based retrospective review of mammograms performed in To-Be 1

The blinded individual review included 239 DBT + SM screening examinations from women screened in To-Be 1 with a false-positive screening result (*n* = 39), interval cancer (*n* = 19), consecutive round screen-detected cancer (*n* = 91), and consecutive round negative screen (*n* = 90) (Fig. 1).

The five external radiologists, not involved in the trials, registered mammographic density (BI-RADS, ad) [20] and described lesions by features (mass, spiculated mass, calcification, asymmetry, architectural distortion, and density with calcification), location in the breast (breast quadrant), visibility on DBT and on SM, and view (craniocaudal, CC, and mediolateral oblique, MLO). The reviewers also marked the conclusion as a malignancy score of 1–5 for DBT and for SM separately on both breasts. They were not asked to classify the cases as false negative, minimal sign, or true.

**Fig. 2:**
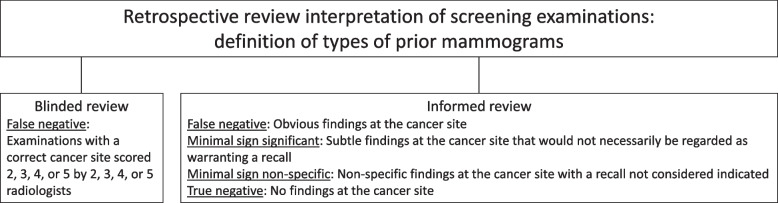
The definition of false negative cases in the blinded individual review and false negative, minimal sign significant, minimal sign non-specific, and true negative cases in the informed consensus-based review for screening examinations prior to interval cancer and consecutive round screen-detected cancer

The definition of false negative was retrospectively created by the authors and included cases with a score of 2 or higher by two or more radiologists (Fig. 2).

### Informed review

After the blinded review, the five radiologists performed the informed consensus-based review (Additional file 1: Appendix B). The review included 110 negative screening examinations of women diagnosed with interval or consecutive round screen-detected cancer (Fig. 1). Prior screening mammograms, diagnostic images, and histopathological findings for all 110 cases were available for the radiologists.

Data recorded at the informed review included mammographic features and conclusion for DBT + SM (false negative, minimal sign significant, minimal sign non-specific, and true negative). If all five radiologists scored 1 for both breasts in the blinded review, the examination was considered true negative, and the images were not reviewed (*n* = 53). If one or more radiologists scored 2 or higher in the blinded review, the examination was discussed (*n* = 57), and it was jointly decided if the cancer was false negative, minimal sign significant, minimal sign non-specific, or true negative. False negative cases were defined as examinations with obvious findings at the cancer site [12, 21] (Fig. 2).

### Reviewers’ characteristics and variables of interest

The reviews were performed by five breast radiologists with the following years of experience in screen reading of DM/reading of DBT: AG 16/9, KL 12/12, RM 10/8, TH 13/10, and SRH 11/11. All images were free from clinical annotations. Data about histopathologic tumor characteristics, including tumor diameter (mm), histologic grade (1–3 by Nottingham scale), lymph node status (positive or negative), estrogen, progesterone and human epidermal growth factor receptor 2 (HER2) status [22], and immunohistochemical subtypes (luminal A, luminal B HER2 − , luminal B HER2 + , HER2 + , and triple negative) [22], were extracted from the cancer registry.

### Statistical analyses

The number and proportion of screening examinations scored 2 or higher in the blinded review were presented for examinations prior to interval cancers and consecutive round screen-detected cancer, by radiologist. Results for examinations with a negative and false positive screening result were shown in the Additional file 1: Table C1. Numbers and proportions of examinations prior to interval or consecutive round screen-detected cancer scored 2 or higher in the blinded review by one or more, two or more, and three or more radiologists were presented for DBT + SM, DBT, and SM. The same results were shown for negative and false positive cases in the Additional file 1: Table C2. The proportions were compared using a chi-square test.

Numbers and proportions of screening examinations prior to interval cancer and consecutive round screen-detected cancer were presented for scores of 2 and 3 or higher by one, two, three, or more radiologists. The same results were shown for true negative and false positive examinations in the Additional file 1: Table C3. The number and proportion of false negatives for screening examinations prior to interval cancer and examinations resulting in consecutive round screen-detected cancer were presented.

Numbers and proportions of false negative, minimal sign significant, minimal sign non-specific, and true negative cases among screening examinations prior to interval and consecutive round screen-detected cancer were shown as assigned in the informed review. These examinations are presented by mammographic density and histopathologic tumor characteristics. A two-sided *p*-value of < 0.05 was considered statistically significant.

## Results

### Blinded individual review

The blinded individual review included 239 DBT + SM screening examinations from To-Be 1 (Fig. 1).

There was substantial variation in interpretation scores across radiologists (Table 1). For example, for screening examinations prior to interval cancer, Radiologist 4 assigned a score of 2, 3, 4, or 5 once (5.3%), while Radiologist 5 assigned those scores to four cases (21.1%). For consecutive round screen-detected cancer, Radiologist 4 scored 2, 3, 4, or 5 in 16 cases (17.6%), while Radiologist 1 assigned those scores to 45 (49.5%) cases.

**Table 1:** Number and proportion of screening examinations with a score of 2, 3, 4, and 5 by radiologists in the blinded individual review of 19 screening examinations prior to interval cancer and 91 screening examinations resulting in consecutive round screen-detected cancer

Proportion assigned	Screening examinations prior to interval cancer (*n* = 19)		Screening examinations resulting in consecutive round screen-detected cancer (*n* = 91)		Total (*n* = 110)	
	*n*	% (fraction)	*n*	% (fraction)	*n*	% (fraction)
**Score 2**						
Radiologist 1	1	5.3 (1/19)	11	12.1 (11/91)	12	10.9 (12/110)
Radiologist 2	0	0	11	12.1 (11/91)	11	10.0 (11/110)
Radiologist 3	0	0	14	15.4 (14/91)	14	12.7 (14/110)
Radiologist 4	1	5.3 (1/19)	14	15.4 (14/91)	15	13.6 (15/110)
Radiologist 5	2	10.5 (2/19)	24	26.4 (24/91)	26	23.6 (26/110)
**Score 3**						
Radiologist 1	1	5.3 (1/19)	20	22.0 (20/91)	21	19.1 (21/110)
Radiologist 2	2	10.5 (2/19)	15	16.5 (15/91)	17	15.5 (17/110)
Radiologist 3	2	10.5 (2/19)	20	22.0 (20/91)	22	20.0 (22/110)
Radiologist 4	0	0	2	2.2 (2/91)	2	1.8 (2/110)
Radiologist 5	2	10.5 (2/19)	9	9.9 (9/91)	11	10.0 (11/110)
**Score 4**						
Radiologist 1	1	5.3 (1/19)	11	12.1 (11/91)	12	10.9 (12/110)
Radiologist 2	1	5.3 (1/19)	5	5.5 (5/91)	6	5.5 (6/110)
Radiologist 3	1	5.3 (1/19)	15	16.5 (15/91)	16	14.5 (16/110)
Radiologist 4	0	0	0	0	0	0
Radiologist 5	0	0	3	3.3 (3/91)	3	2.7 (3/110)
**Score 5**						
Radiologist 1	0	0	3	3.3 (3/91)	3	2.7 (3/110)
Radiologist 2	0	0	3	3.3 (3/91)	3	2.7 (3/110)
Radiologist 3	0	0	3	3.3 (3/91)	3	2.7 (3/110)
Radiologist 4	0	0	0	0	0	0
Radiologist 5	0	0	0	0	0	0

For the negative examinations, the variation of the number of cases assigned with a score of 2 ranged between 10 (11.1%) and 24 (26.7%) (Additional file 1: Table C1). For screening examinations with a false positive result, the number of cases varied from 10 (25.6%) to 26 (66.7%).

For screening examinations resulting in consecutive round screen-detected cancer (*n* = 91), the proportion of a score of 4 or 5 by one or more radiologists was 30.8% for DBT alone versus 8.8% for SM alone (*p* < 0.001) (Table 2). The proportion of a score of 3 by two or more radiologists was 16.6% for DBT alone versus 5.5% for SM alone (*p* = 0.02). Results for negative and false positive screening results were shown in the (Additional file 1: Table C2).

**Table 2:** Number^a^ and proportion of 19 screening examinations prior to interval cancer and 91 screening examinations resulting in consecutive round screen-detected cancer, scored 2, 3, and 4 or 5 by one or more, two or more, and three or more of the five radiologists for digital breast tomosynthesis (DBT) + synthetic 2D images (SM), DBT alone, and SM alone in the blinded individual review

**Screening examinations prior to interval cancer (*n* = 19)**	**DBT + SM**		**DBT**		**SM**	
Proportion assigned in a review	*n*	% (fraction)	*n*	% (fraction)	*n*	% (fraction)
Score 2 by 1 or more radiologists	3	15.8 (3/19)	2	10.5 (2/19)	2	10.5 (2/19)
Score 3 by 1 or more radiologists	4	21.5 (4/19)	3	15.8 (3/19)	4	21.5 (4/19)
Score 4 or 5 by 1 or more radiologists	2	10.5 (2/19)	2	10.5 (2/19)	0	0
Score 2 by 2 or more radiologists	1	5.3 (1/19)	0	0	1	5.3 (1/19)
Score 3 by 2 or more radiologists	2	10.5 (2/19)	1	5.3 (1/19)	1	5.3 (1/19)
Score 4 or 5 by 2 or more radiologists	1	5.3 (1/19)	1	5.3 (1/19)	0	0
Score 2 by 3 or more radiologists	0	0	0	0	0	0
Score 3 by 3 or more radiologists	1	5.3 (1/19)	1	5.3 (1/19)	0	0
Score 4 or 5 by 3 or more radiologists	0	0	0	0	0	0
**Screening examinations resulting in consecutive round screen-detected cancer (*n* = 91)**	**DBT + SM**		**DBT**		**SM**	
Proportion assigned in a review	*n*	% (fraction)	*n*	% (fraction)	*n*	% (fraction)
Score 2 by 1 or more radiologists	43	47.3 (43/91)	35	38.5 (35/91)	34	37.4 (34/91)
Score 3 by 1 or more radiologists	41	45.1 (41/91)	34	37.4 (34/91)	23	25.3 (23/91)
Score 4 or 5 by 1 or more radiologists	33	36.3 (33/91)	28	30.8 (28/91)	8	8.8^*^ (8/91)
Score 2 by 2 or more radiologists	22	24.2 (22/91)	12	13.2 (12/91)	12	13.2 (12/91)
Score 3 by 2 or more radiologists	18	19.9 (18/91)	15	16.5 (15/91)	5	5.5^**^ (5/91)
Score 4 or 5 by 2 or more radiologists	6	6.6 (6/91)	6	6.6 (6/91)	1	1.1 (1/91)
Score 2 by 3 or more radiologists	7	7.7 (7/91)	3	3.3 (3/91)	4	4.4 (4/91)
Score 3 by 3 or more radiologists	7	7.7 (7/91)	4	4.4 (4/91)	3	3.3 (3/91)
Score 4 or 5 by 3 or more radiologists	3	3.3 (3/91)	3	3.3 (3/91)	0	0

Score 2 probably benign, 3 intermediate suspicion of malignancy, 4 probably malignant, and 5 high suspicion of malignancy

^*^p < 0.001 for comparison of DBT and SM

^**^p = 0.02 for comparison of DBT and SM

^a^Number shows the number of cases chosen/assigned for each radiologist and different combinations of radiologists, some of these cases are the same, but the cases could also differ for the different radiologists

Screening examinations considered false negative after the blinded review (assigned with a score of 2, 3, 4, or 5 by two, three, four, or five radiologists) included 10.5% (2/19) of examinations prior to interval cancer and 42.9% (39/91) of examinations resulting in consecutive round screen-detected cancer. A score of 2, 3, 4, or 5 by two, three, four, or five radiologists was also assigned to 47.8% (43/90) of negative examinations and 89.7% (35/39) of examinations with a false positive result (Table 3 and Additional file 1: Table C3).

**Table 3:** Number and proportion of screening examinations prior to interval cancer and examinations resulting in consecutive round screen-detected cancer for a score of 2 or higher and a score of 3 or higher by one or more radiologists, two or more radiologists, and three or more radiologists

	**Screening examinations prior to interval cancer (*n* = 19)**		**Screening examinations resulting in consecutive round screen-detected cancer (*n* = 91)**		**Total (*n* = 110)**	
	*n*	% (fraction)	*n*	% (fraction)	*n*	% (fraction)
Scores 2, 3, 4, or 5 by 1, 2, 3, 4, or 5 radiologists	5	26.3% (5/19)	52	57.1% (52/91)	57	51.8% (57/110)
**Scores 2, 3, 4, or 5 by 2, 3, 4, or 5 radiologists**	**2**	**10.5% (2/19)**	**39**	**42.9% (39/91)**	**41**	**37.3% (41/110)**
Scores 2, 3, 4, or 5 by 3, 4, or 5 radiologists	2	10.5% (2/19)	25	27.5% (25/91)	27	24.5% (27/110)
Scores 3, 4, or 5 by 1, 2, 3, 4, or 5 radiologists	4	21.1% (4/19)	46	50.5% (46/91)	50	45.5% (50/110)
Scores 3, 4, or 5 by 2, 3, 4, or 5 radiologists	2	10.5% (2/19)	26	28.6% (26/91)	28	25.5% (28/110)
Scores 3, 4, or 5 by 3, 4, or 5 radiologists	1	5.3% (1/19)	14	15.4% (14/91)	15	13.6% (15/110)

### Informed consensus-based review

The informed review included a total of 110 DBT + SM screening examinations prior to diagnosis of interval cancer (*n* = 19) and consecutive round screen-detected cancer (*n* = 91).

A total of 5.3% (1/19) of the interval cancers were considered false negative, 10.5% (2/19) minimal sign significant, 10.5% (2/19) minimal sign non-specific, and 73.4% (14/19) true negative (Table 4). For screening examinations resulting in consecutive round screen-detected cancer, 18.7% (17/91) were assigned as false negative, 15.4% (14/91) as minimal sign significant, 15.4% (14/91) as minimal sign non-specific, and 50.6% (46/91) as true negative. The total number of screening examinations assigned as false negative, interval plus consecutive round screen-detected cancer, was 16.4% (18/110).

**Table 4:** Number and percentage of false negative, minimal sign significant, minimal sign non-specific, and true cancers on screening examinations prior to interval cancer (*n* = 19) and resulting in consecutive round screen-detected cancer (*n* = 91) based on the informed consensus-based review

	Screening examinations prior to interval cancer (*n* = 19)		Screening examinations resulting in consecutive round screen-detected cancer (*n* = 91)		Total (*n* = 110)	
	*n*	% (fraction)	*n*	% (fraction)	*n*	% (fraction)
False negative	1	5.3 (1/19)	17	18.7 (17/91)	18	16.4 (18/110)
Minimal sign significant	2	10.5 (2/19)	14	15.4 (14/91)	16	14.5 (16/110)
Minimal sign non-specific	2	10.5 (2/19)	14	15.4 (14/91)	16	14.5 (16/110)
True	14	73.7 (14/19)	46	50.6 (46/91)	60	54.6 (60/110)

Among examinations resulting in consecutive round screen-detected cancer and assigned as false negative, 76% (13/17) had BI-RADS density c, 35% (6/17) lesions were architectural distortion, 88% (15/17) were invasive cancers, 67% (10/15) had tumor diameter < 21 mm, 67% (10/15) had histologic grades 1 or 2, and 60% (9/15) had luminal A immunohistochemical subtype (Table 5).

**Table 5:** Distribution of screening examinations prior to interval cancer after To-Be 1 (*n* = 19) and resulting in consecutive round screen-detected cancer in To-Be 2 (*n* = 91) classified into false negative, minimal sign significant, minimal sign non-specific, and true negative in the informed consensus-based review, by mammographic density and histopathologic tumor characteristics

	Screening examinations prior to interval cancer (*n* = 19)								Screening examinations resulting in consecutive round screen-detected cancer (*n* = 91)							
	False negative		Minimal sign significant		Minimal sign non-specific		True negative		False negative		Minimal sign significant		Minimal sign non-specific		True negative	
***n***	**1**		**2**		**2**		**14**		**17**		**14**		**14**		**46**	
**%**	**5%**		**11%**		**11%**		**74%**		**19%**		**15%**		**15%**		**51%**	
Mammographic density																
a	0	0%	0	0%	0	0%	0	0%	0	0%	0	0%	3	21%	2	4%
b	1	100%	0	0%	0	0%	1	7%	3	18%	5	36%	2	14%	14	30%
c	0	0%	2	100%	2	100%	8	57%	13	76%	4	29%	6	43%	22	48%
d	0	0%	0	0%	0	0%	5	36%	1	6%	5	36%	3	21%	8	17%
Mammographic feature																
Mass	0	0%	0	0%	1	50%	-		3	18%	4	29%	2	14%	-	
Spiculated mass	0	0%	0	0%	0	0%	-		3	18%	2	14%	2	14%	-	
Asymmetry	0	0%	1	50%	0	0%	-		2	12%	0	0%	3	21%	-	
Architectural distortion	0	0%	0	0%	1	50%	-		6	35%	4	29%	3	21%	-	
Calcification	0	0%	0	0%	0	0%	-		1	6%	2	14%	4	29%	-	
Density with calcification	1	100%	1	50%	0	0%	-		2	12%	2	14%	0	0%	-	
Type																
Ductal carcinoma in situ	0	0%	0	0%	0	0%	0	0%	2	12%	2	14%	5	36%	5	11%
Invasive	1	100%	2	100%	2	100%	14	100%	15	88%	12	86%	9	64%	41	89%
No special type	0	0%	1	50%	1	50%	10	71%	5	33%	10	83%	7	78%	30	73%
Lobular	0	0%	0	0%	0	0%	2	14%	5	33%	1	8%	1	11%	8	20%
Tubular	0	0%	0	0%	1	50%	0	0%	5	33%	0	0%	1	11%	0	0%
Other	1	100%	1	50%	0	0%	2	14%	0	0%	1	8%	0	0%	3	7%
Tumor diameter																
≤ 10 mm	0	-	0	0%	1	50%	0	0%	6	43%	4	36%	2	22%	10	29%
11–20 mm	0	-	0	0%	0	0%	6	75%	4	29%	7	64%	6	67%	17	50%
> 20 – ≤ 50 mm	0	-	1	100%	1	50%	2	25%	4	29%	0	0%	1	11%	7	21%
*Missing*^a^	*1*		*1*		*0*		*6*		*1*		*1*		*0*		*7*	
Histologic grade																
Grade 1	0	-	0	0%	1	50%	1	8%	6	40%	7	64%	1	11%	14	39%
Grade 2	0	-	0	0%	0	0%	7	58%	4	27%	4	36%	6	67%	16	44%
Grade 3	0	-	1	100%	1	50%	4	33%	5	33%	0	0%	2	22%	6	17%
* Missing*	*1*		*1*		*0*		*2*		*0*		*1*		*0*		*5*	
Lymph node positive	0	0%	0	0%	0	0%	6	43%	2	13%	0	0%	1	11%	4	10%
Subtype																
Luminal A	0	0%	0	0%	1	50%	4	29%	9	60%	9	82%	5	56%	24	60%
Luminal B HER2 −	0	0%	0	0%	0	0%	5	36%	6	40%	2	18%	2	22%	10	25%
Luminal B HER2 +	1	100%	0	0%	0	0%	2	14%	0	0%	0	0%	1	11%	3	8%
HER2 +	0	0%	1	50%	0	0%	1	7%	0	0%	0	0%	1	11%	1	3%
Triple negative	0	0%	1	50%	1	50%	2	14%	0	0%	0	0%	0	0%	2	5%
*Missing*	*0*		*0*		*0*		*0*		*0*		*1*		*0*		*1*	

*HER2* human epidermal growth factor receptor 2

^a^Locally advanced

## Discussion

As far as we are aware, no prior studies have performed blinded and informed review of prior DBT images to classify cancer cases as false or true negative. According to our definitions, 10.5% (2/19) of the screening examinations prior to interval cancer, 42.9% (39/91) of the screening examinations resulting in consecutive round screen-detected cancer from the To-Be 1 trial were scored ≥ 2 and classified as false negative after the blinded individual review. The same score (≥ 2) was assigned to 47.8% (43/90) of the negative and 89.7% (35/39) of the false positive examinations. The informed consensus-based review by five experienced breast radiologists not involved in the To-Be trials classified 5.3% (1/19) of the screening examinations prior to interval cancer and 18.7% (17/91) of screening examinations resulting in consecutive round screen-detected cancer as false negatives.

As expected, the malignant lesions were more frequently visible on DBT compared with SM, specifically for examinations resulting in consecutive screen-detected cancer scored 4 or 5 by one or more radiologists in the blinded review [23, 24]. Previous studies have shown that small-detail detectability could be reduced for SM compared to DBT, specifically for detection of the desmoplastic processes associated with spiculated masses and architectural distortions visible solely on one or few DBT planes, but not on SM [25, 26]. However, these findings could not corroborate any assumptions on increased breast cancer detection for DBT versus DM or SM in To-Be 1. Moreover, the proportion of negative examinations and examinations with a false positive screening result scored ≥ 3 by one or more radiologists was significantly higher for DBT compared to SM, implying that DBT may be associated with an increased rate of false positives [27, 28].

Prognostically favorable histopathologic tumor characteristics of the examinations resulting in consecutive round screen-detected cancers classified as false negative in the informed review suggest that earlier detection of these cancers would be of limited clinical value. Moreover, only 1 of the 19 screening examinations resulting in interval cancer was classified as false negative in the informed review, indicating fast growing tumors among the interval cancers [12, 29–32].

Previous DBT interpretive studies have not assessed the rates of false negative cases [33–35]. Informed review studies performed on DM and screen-film mammography (SFM) have reported a false negative rate varying from 12 to 36%, including DM studies from BreastScreen Norway showing 19–34% of false negatives for examinations prior to interval cancers and 20–22% of false negatives for examinations resulting in consecutive round screen-detected cancers [10–18]. The lower percentage of false negatives for examinations prior to interval cancers in our blinded and informed review compared to the results of prior studies using DM and SFM may be due to the low number of study cases. Furthermore, the percentage of false negatives has been reported to be influenced by the comparability and similarity between the study setting and a normal screening setting [12].

### Strengths and limitations

Strengths of our design included the reviews being conducted by five external breast radiologists, not involved in the To-Be trials, reducing the risk of bias associated with the interpretation. The blinded review set included negative and false positive examinations simulating a normal screening setting. Finally, our data were from a population-based breast cancer screening program with a high completeness of histologically verified breast cancer cases.

However, different review procedures used in the blinded individual and informed consensus-based review limited the possibility to compare or combine the numbers and proportions of false negatives. There were large variations in results for scores ≥ 2, ≥ 3, etc. by different combinations of radiologists, including high percentages of scores ≥ 2 and ≥ 3 for negative screening examinations, which restricted our definition of false negatives in the blinded review. The blinded review was, however, important for the external radiologists in terms of familiarization with the dataset and showed large individual differences in the interpretation, which most likely arose due to different classification systems in other countries and/or image quality for DBT systems. Nevertheless, BreastScreen Norway uses independent double reading with consensus with a recall solely assigned by consensus consisting of at least two radiologists, and we assume our definition of false negatives in the blinded review led to somewhat overestimated results but was analogous to the possibility of concordant choice of a score of 2 or higher by at least two radiologists [12]. The presence of two minimal sign categories in the informed review might have led to a lower number of false negatives versus use of one category only. The percentage of false negatives identified by the external reviewers in consensus could be a result of an experimental effect of reduced specificity inherent to the informed review methodology [17]. Furthermore, as image quality is an important aspect for breast cancer detection [36, 37], the external radiologists questioned the technical image quality in To-Be 1, performed with GE SenoClaire. The imaging equipment might be of influence for the low number of false negative cases in the informed review. The image quality may therefore represent one possible reason for the low rate of false negative examinations in the informed review but also for the lack of expected increase in cancer detection for DBT. The radiologists stated that a substantially better quality was observed for GE Pristina; however, no objective measurements of the image quality were collected; therefore, we were not able to draw any conclusions. Moreover, the postprocessing or reconstruction of study images was not performed. The experience with DBT for the external radiologists could have been associated with different vendors compared to those used in the study, which might have affected the ability to detect suspicious lesions. The large differences in percentages of examinations resulting in cancer, as well as false positive and negative examinations, scored ≥ 2 by the external readers might underline the uncertainty in the obtained results. It is also possible that To-Be 2 diagnosed more screen-detected cancers due to use of DBT + SM as the only screening technique for all women, which might have resulted in the overestimation of the basis for false negative cases among screen-detected cancers in our study, for both the DBT- and DM-arm.

Finally, this review did not include images from the DM-arm. However, blinded and informed reviews on DM and SFM have been performed with comparable results as in this DBT review [10–14, 17, 38, 39]. Results from a mixed blinded individual review of interval cancers from SFM performed in 2005 showed that 20% (46 of 231) of cases were false negative [13], while another blinded review of screening mammograms 2 and 4 years prior examinations of screen-detected cancer showed that 31% (32 of 103) of cases were false negative [38]. These numbers can be used to calculate the rates of false negatives in the potential blinded review of the DM-arm of the To-Be 1 trial. In the DM-arm, the original number of screen-detected cancers was 87 [7], and the number of interval cancers was 29, while the number of subsequent screen-detected cancers following the DM-arm was 101 [8]. Therefore, the number of false negatives resulting in interval cancers in the DM-arm could have been about 6 (29 × 20/100) and the number of false negative screen-detected cancers about 31 (101 × 31/100) in the potential blinded review. This would have resulted in 37 extra cases (false negatives) of screen-detected cancers in the DM-arm, accounting for 124 (87 + 37) in total, and a rate of 0.86%. When including the results from our blinded review on the number of false negatives for the DBT + SM-arm (41 cases), a total of 136 (95 + 41) in 14,380 women for DBT + SM corresponds to a detection rate of 0.95%, versus 0.86% in the DM-arm (124 cases in 14,369 women), and a *p*-value of 0.42.

Results from two informed consensus-based reviews of DM examinations from 2004 to 2016 BreastScreen Norway, including 24% of false negatives for interval cancer (246 of 1010) and 22% of false negatives for consecutive round screen-detected breast cancer (266 of 1225) [11, 12], can also be used to estimate the number of potential false negatives in the DM-arm of the To-Be 1 in the informed review. The number of false negative cases resulting in interval cancers in the DM-arm would have been about 7 (29 × 24/100) and the number of false negative screen-detected cancers about 22 (101 × 22/100). This would have resulted in 29 extra cases (false negatives) of screen-detected cancers in the DM-arm, accounting for 116 (87 + 29) in total and a rate of 0.81%. When including the results from our informed review on the number of false negatives for the DBT + SM-arm (18 cases), a total of 113 (95 + 18) in 14,380 women for DBT + SM corresponds to a rate of screen-detected cancer of 0.79%, versus 0.81% in the DM-arm (116 cases in 14,369 women, *p* = 0.79) [7]. Under these assumptions, the DBT + SM versus DM cancer detection rate would not differ statistically significantly. However, these findings should be interpreted with caution. The number of false negatives in the DM-arm may have been overestimated, as DBT + SM was used to detect breast cancer in the follow-up of the DM-arm and the rates of false negatives in the DM-arm were calculated based on the data from 1990s to 2016 from different countries and programs [13, 14, 38].

In conclusion, this study examined potential false negative interval and consecutive round screen-detected cancers in the To-Be 1 trial and demonstrated that the percentages determined by both individual and consensus expert reviews were consistent with prior DM review studies. The results of this review indicate that the nonsignificant difference in cancer detection between DBT + SM versus DM in the To-Be 1 trial is complex and not caused by interpretive error alone.

### Supplementary Information

**Additional file 1: Appendix A.** Form for the blinded review. **Appendix B.** Form for the informed consensus review. **Appendix C.** Table C1. Number and proportion of screening examinations with a score of 2, 3, 4, and 5 by radiologists in the individual blinded review of 90 negative screening examinations in To-Be 1 and To-Be 2 and 39 screening examinations with a false positive screening result. Table C2. Number^#^ and proportion of 90 true negative screening examinations and 39 screening examinations with a false positive screening result, scored 2, 3, and 4 or 5 by one or more, two or more, and three or more radiologists for digital breast tomosynthesis (DBT) + synthetic 2D images (SM), DBT alone and SM alone in the individual blinded review. Score 2: probably benign, 3: intermediate suspicion of malignancy, 4: probably malignant, and 5: high suspicion of malignancy. Table C3. Number and proportion of negative screening examinations, and examinations with a false positive result for a score of 2 or higher, and a score of 3 or higher by one or more radiologists, two or more radiologists, and three or more radiologists.

## Data Availability

Data used in the analyses can be made available on request to https://helsedata.no/, given legal basis in Articles 6 and 9 of the GDPR, and that the processing is in accordance with Article 5 of the GDPR.

## Abbreviations

BI-RADS: Breast Imaging-Reporting and Data System
CC: Craniocaudal
DBT + SM: Digital breast tomosynthesis and synthetic 2D mammograms
DM: Digital mammography
HER2: Human epidermal growth factor receptor 2
MLO: Mediolateral oblique
RCT: Randomized controlled trial
SFM: Screen-film mammography

## References

[1] Heindel W, Weigel S, Gerß J (2022). Digital breast tomosynthesis plus synthesised mammography versus digital screening mammography for the detection of invasive breast cancer (TOSYMA): a multicentre, open-label, randomised, controlled, superiority trial. Lancet Oncol.

[2] Pattacini P, Nitrosi A, Giorgi Rossi P (2022). A randomized trial comparing breast cancer incidence and interval cancers after tomosynthesis plus mammography versus mammography alone. Radiology.

[3] Caumo F, Zorzi M, Brunelli S (2018). Digital breast tomosynthesis with synthesized two-dimensional images versus full-field digital mammography for population screening: outcomes from the Verona screening program. Radiology.

[4] Bernardi D, Macaskill P, Pellegrini M (2016). Breast cancer screening with tomosynthesis (3D mammography) with acquired or synthetic 2D mammography compared with 2D mammography alone (STORM-2): a population-based prospective study. Lancet Oncol.

[5] Houssami N, Zackrisson S, Blazek K, Hunter K, Bernardi D, Lång K (2021). Meta-analysis of prospective studies evaluating breast cancer detection and interval cancer rates for digital breast tomosynthesis versus mammography population screening. Eur J Cancer.

[6] Johnson K, Lång K, Ikeda DM, Åkesson A, Andersson I, Zackrisson S (2021). Interval breast cancer rates and tumor characteristics in the prospective population-based Malmö Breast Tomosynthesis Screening Trial. Radiology.

[7] Hofvind S, Holen ÅS, Aase HS (2019). Two-view digital breast tomosynthesis versus digital mammography in a population-based breast cancer screening programme (to-be): a randomised, controlled trial. Lancet Oncol.

[8] Hofvind S, Moshina N, Holen ÅS (2021). Interval and subsequent round breast cancer in a randomized controlled trial comparing digital breast tomosynthesis and digital mammography screening. Radiology.

[9] Zackrisson S (2019). Tomosynthesis in breast screening: great expectations?. Lancet Oncol.

[10] Ciatto S, Rosselli Del Turco M, Zappa M (1995). The detectability of breast cancer by screening mammography. Br J Cancer.

[11] Hovda T, Tsuruda K, Hoff SR, Sahlberg KK, Hofvind S (2021). Radiological review of prior screening mammograms of screen-detected breast cancer. Eur Radiol.

[12] Hovda T, Hoff SR, Larsen M, Romundstad L, Sahlberg KK, Hofvind S (2022). True and missed interval cancer in organized mammographic screening: a retrospective review study of diagnostic and prior screening mammograms. Acad Radiol.

[13] Hofvind S, Skaane P, Vitak B (2005). Influence of review design on percentages of missed interval breast cancers: retrospective study of interval cancers in a population-based screening program. Radiology.

[14] Hoff SR, Abrahamsen A-L, Samset JH, Vigeland E, Klepp O, Hofvind S (2012). Breast cancer: missed interval and screening-detected cancer at full-field digital mammography and screen-film mammography— results from a retrospective review. Radiology.

[15] van Dijck JA, Verbeek AL, Hendriks JH, Holland R (1993). The current detectability of breast cancer in a mammographic screening program. A review of the previous mammograms of interval and screen-detected cancers. Cancer.

[16] Moberg K, Grundström H, Törnberg S (1999). Two models for radiological reviewing of interval cancers. J Med Screen.

[17] Ciatto S, Catarzi S, Lamberini MP (2007). Interval breast cancers in screening: the effect of mammography review method on classification. Breast.

[18] de Rijke JM, Schouten LJ, Schreutelkamp JL, Jochem I, Verbeek AL (2000). A blind review and an informed review of interval breast cancer cases in the Limburg screening programme, the Netherlands. J Med Screen.

[19] Bjørnson EW, Holen ÅS, Sagstad S, Larsen M, Thy J, Mangerud G (2022). BreastScreen Norway: 25 years of organized screening.

[20] Sickles E, D’Orsi CJ, Bassett LW, et al (2013) ACR BI-RADS® mammography. ACR BI-RADS® Atlas, Breast Imaging Reporting and Data System, 5th edn. American College of Radiology, Reston

[21] Perry N, Broeders M, de Wolf C, Törnberg S, Holland R, von Karsa L (2006). European Commission. European guidelines for quality assurance in breast cancer screening and diagnosis.

[22] Falck AK, Rome A, Ferno M (2016). St Gallen molecular subtypes in screening-detected and symptomatic breast cancer in a prospective cohort with long-term follow-up. Br J Surg.

[23] Alabousi M, Wadera A, Kashif Al-Ghita M (2021). Performance of digital breast tomosynthesis, synthetic mammography, and digital mammography in breast cancer screening: a systematic review and meta-analysis. J Natl Cancer Inst.

[24] Abdullah P, Alabousi M, Ramadan S (2021). Synthetic 2D mammography versus standard 2D digital mammography: a diagnostic test accuracy systematic review and meta-analysis. AJR Am J Roentgenol.

[25] Vancoillie L, Cockmartin L, Marshall N, Bosmans H (2021). The impact on lesion detection via a multi-vendor study: a phantom-based comparison of digital mammography, digital breast tomosynthesis, and synthetic mammography. Med Phys.

[26] Skaane P (2023). Interval and successive-round cancers after digital breast tomosynthesis screening: we still need convincing results regarding beneficial evidence on long-term outcomes. Radiology.

[27] Zuckerman SP, Sprague BL, Weaver DL, Herschorn SD, Conant EF (2020). Multicenter evaluation of breast cancer screening with digital breast tomosynthesis in combination with synthetic versus digital mammography. Radiology.

[28] Sujlana PS, Mahesh M, Vedantham S, Harvey SC, Mullen LA, Woods RW (2019). Digital breast tomosynthesis: image acquisition principles and artifacts. Clin Imaging.

[29] Tsuruda KM, Hovda T, Bhargava S, Veierød MB, Hofvind S (2021). Survival among women diagnosed with screen-detected or interval breast cancer classified as true, minimal signs, or missed through an informed radiological review. Eur Radiol.

[30] Houssami N, Bernardi D, Caumo F, Brunelli S, Fantò C, Valentini M (2018). Interval breast cancers in the ‘screening with tomosynthesis or standard mammography’ (STORM) population-based trial. Breast.

[31] Groenendijk RP, Bult P, Noppen CM, Boetes C, Ruers TJ, Wobbes T (2003). Mitotic activity index in interval breast cancers. Eur J Surg Oncol.

[32] Duncan AA, Wallis MG (1995). Classifying interval cancers. Clin Radiol.

[33] Rodriguez-Ruiz A, Gubern-Merida A, Imhof-Tas M (2018). One-view digital breast tomosynthesis as a stand-alone modality for breast cancer detection: do we need more?. Eur Radiol.

[34] Chan HP, Helvie MA, Hadjiiski L (2017). Characterization of breast masses in digital breast tomosynthesis and digital mammograms: an observer performance study. Acad Radiol.

[35] Durand MA, Wang S, Hooley RJ, Raghu M, Philpotts LE (2016). Tomosynthesis-detected architectural distortion: management algorithm with radiologic-pathologic correlation. Radiographics.

[36] Seely JM, Eby PR, Yaffe MJ (2022). The fundamental flaws of the CNBSS trials: a scientific review. J Breast Imaging.

[37] Rauscher GH, Conant EF, Khan JA, Berbaum ML (2013). Mammogram image quality as a potential contributor to disparities in breast cancer stage at diagnosis: an observational study. BMC Cancer.

[38] Maxwell AJ (1999). Breast cancers missed in the prevalent screening round: effect upon the size distribution of incident round detected cancers. J Med Screen.

[39] Daly CA, Apthorp L, Field S (1998). Second round cancers: how many were visible on the first round of the UK National Breast Screening Programme, three years earlier?. Clin Radiol.

