# Supplementary material for: Digital breast tomosynthesis in mammographic screening: false negative cancer cases in the To-Be 1 trial
Source: Insights Imaging. 2024 Feb 8;15:38. doi: 10.1186/s13244-023-01604-5 (PMC10853101; doi:10.1186/s13244-023-01604-5)
Supplement: Supplementary file 1 — Additional file 1: Appendix A. Form for the blinded review. Appendix B. Form for the informed consensus review. Appendix C. Table C1. Number and proportion of screening examinations with a score of 2, 3, 4, and 5 by radiologists in the individual blinded review of 90 negative screening examinations in To-Be 1 and To-Be 2 and 39 screening examinations with a false positive screening result. Table C2. Number# and proportion of 90 true negative screening examinations and 39 screening examinations with a false positive screening result, scored 2, 3, and 4 or 5 by one or more, two or more, and three or more radiologists for digital breast tomosynthesis (DBT) + synthetic 2D images (SM), DBT alone and SM alone in the individual blinded review. Score 2: probably benign, 3: intermediate suspicion of malignancy, 4: probably malignant, and 5: high suspicion of malignancy. Table C3. Number and proportion of negative screening examinations, and examinations with a false positive result for a score of 2 or higher, and a score of 3 or higher by one or more radiologists, two or more radiologists, and three or more radiologists. [file 13244_2023_1604_MOESM1_ESM.docx]

**Digital Breast Tomosynthesis in mammographic screening: False negative cancer cases in the To-Be 1 trial**

**ELECTRONIC SUPPLEMENTARY MATERIAL**

**Appendix A:** Form for the blinded review
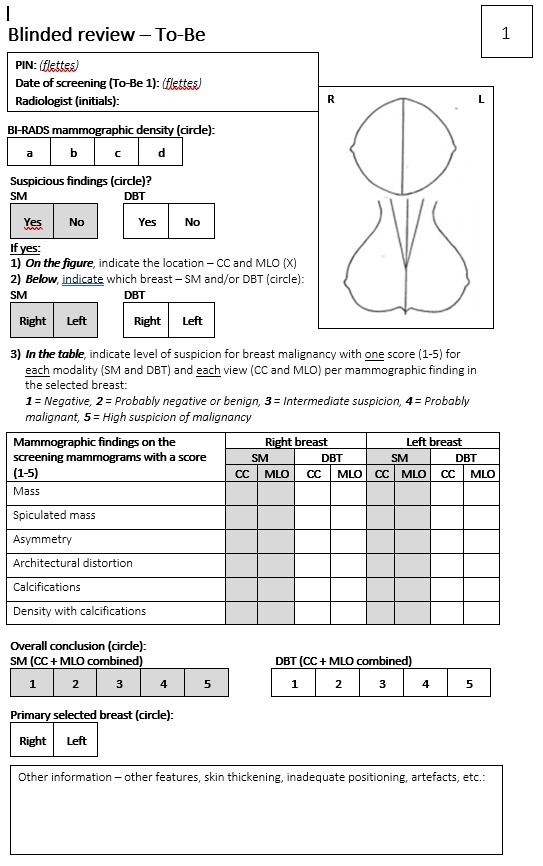


**Appendix B: Form for the informed consensus review**


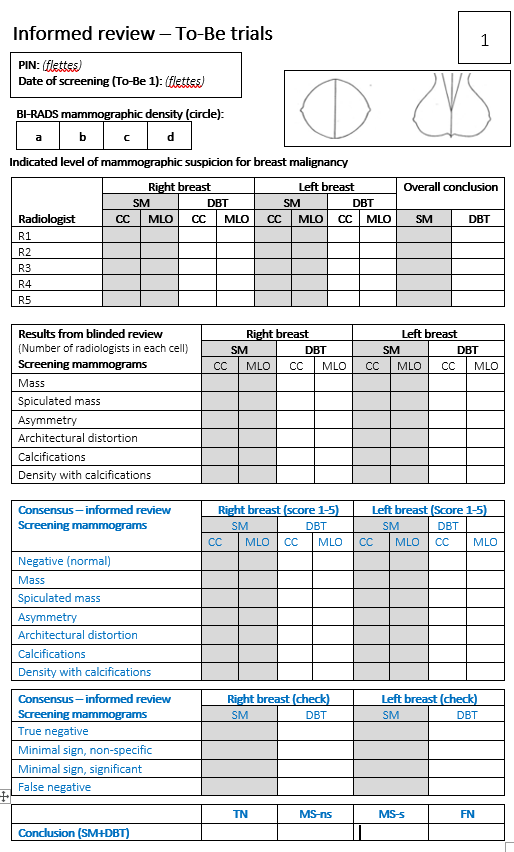


**Appendix C**

Table C1. Number and proportion of screening examinations with a score of 2, 3, 4, and 5 by radiologists in the individual blinded review of 90 negative screening examinations in To-Be 1 and To-Be 2 and 39 screening examinations with a false positive screening result

|  | **Negative screening examinations in To-Be 1 and To-Be 2 (n=90)** | | **Screening examinations with a false positive screening result in To-Be 1, no cancer in To-Be 2 (n=39)** | | Total (n=129) | |
| --- | --- | --- | --- | --- | --- | --- |
| Proportion assigned | n | % (fraction) | n | % (fraction) | n | % (fraction) |
| **Score 2** |  |  |  |  |  |  |
| Radiologist 1 | 18 | 20.0 (18/90) | 10 | 25.6 (10/39) | 28 | 21.7 (28/129) |
| Radiologist 2 | 23 | 25.6 (23/90) | 12 | 30.8 (12/39) | 35 | 27.3 (35/129) |
| Radiologist 3 | 20 | 22.2 (20/90) | 17 | 43.6 (17/39) | 37 | 28.7 (37/129) |
| Radiologist 4 | 10 | 11.1 (10/90) | 19 | 48.7 (19/39) | 29 | 22.5 (29/129) |
| Radiologist 5 | 24 | 26.7 (24/90) | 26 | 66.7 (26/39) | 50 | 38.8 (50/129) |
| **Score 3** |  | |  | |  |  |
| Proportion assigned | n | % (fraction) | n | % (fraction) | n | % (fraction) |
| Radiologist 1 | 11 | 12.2 (11/90) | 15 | 38.5 (15/39) | 26 | 20.2 (26/129) |
| Radiologist 2 | 11 | 12.2 (11/90) | 16 | 41.0 (16/39) | 27 | 20.9 (27/129) |
| Radiologist 3 | 20 | 22.2 (20/90) | 22 | 56.4 (22/39) | 42 | 32.6 (42/129) |
| Radiologist 4 | 1 | 1.1 (1/90) | 1 | 2.6 (1/39) | 2 | 1.6 (2/129) |
| Radiologist 5 | 5 | 5.6 (5/90) | 6 | 15.4 (6/39) | 11 | 8.5 (11/129) |
| **Score 4** |  | |  | |  |  |
| Proportion assigned | n | % (fraction) | n | % (fraction) | n | % (fraction) |
| Radiologist 1 | 1 | 1.1 (1/90) | 3 | 7.7 (3/39) | 4 | 3.1 (4/129) |
| Radiologist 2 | 2 | 2.2 (2/90) | 6 | 15.4 (6/39) | 8 | 6.2 (8/129) |
| Radiologist 3 | 7 | 7.8 (7/90) | 0 | 0.0 | 7 | 5.4 (7/129) |
| Radiologist 4 | 0 | 0.0 | 0 | 0.0 | 0 | 0.0 |
| Radiologist 5 | 0 | 0.0 | 2 | 5.1 (2/39) | 2 | 1.6 (2/129) |
| **Score 5** |  | |  | |  |  |
| Proportion assigned | n | % (fraction) | n | % (fraction) | n | % (fraction) |
| Radiologist 1 | 0 | 0.0 | 2 | 5.1 (2/39) | 2 | 1.6 (2/129) |
| Radiologist 2 | 0 | 0.0 | 1 | 2.6 (1/39) | 1 | 0.8 (1/129) |
| Radiologist 3 | 0 | 0.0 | 0 | 0.0 | 0 | 0.0 |
| Radiologist 4 | 0 | 0.0 | 0 | 0.0 | 0 | 0.0 |
| Radiologist 5 | 0 | 0.0 | 0 | 0.0 | 0 | 0.0 |

Table C2. Number^#^ and proportion of 90 true negative screening examinations and 39 screening examinations with a false positive screening result, scored 2, 3, and 4 or 5 by one or more, two or more, and three or more radiologists for digital breast tomosynthesis (DBT) + synthetic 2D images (SM), DBT alone and SM alone in the individual blinded review. Score 2: probably benign, 3: intermediate suspicion of malignancy, 4: probably malignant, and 5: high suspicion of malignancy.

| **Negative screening examinations in To-Be 1 and To-Be 2 (n=90)** | **DBT+SM** | | **DBT** | | **SM** | |
| --- | --- | --- | --- | --- | --- | --- |
| Proportion assigned in a review | n | % (fraction) | n | % (fraction) | n | % (fraction) |
| Score 2 by 1 or more radiologists | 59 | 65.6 (59/90) | 51 | 56.7 (51/90) | 37 | 41.1* (37/90) |
| Score 3 by 1 or more radiologists | 38 | 42.2 (38/90) | 34 | 37.8 (34/90) | 13 | 14.4** (13/90) |
| Score 4 or 5 by 1 or more radiologists | 11 | 12.2 (11/90) | 9 | 10.0 (9/90) | 2 | 2.2 (2/90) |
|  |  |  |  |  |  |  |
| Score 2 by 2 or more radiologists | 27 | 30.0 (27/90) | 16 | 17.8 (16/90) | 9 | 10.0 (9/90) |
| Score 3 by 2 or more radiologists | 9 | 10.0 (9/90) | 6 | 6.7 (6/90) | 1 | 1.1 (1/90) |
| Score 4 or 5 by 2 or more radiologists | 1 | 1.1 (1/90) | 1 | 1.1 (1/90) | 0 | 0 |
|  |  |  |  |  |  |  |
| Score 2 by 3 or more radiologists | 6 | 6.7 (6/90) | 3 | 3.3 (3/90) | 3 | 3.3 (3/90) |
| Score 3 by 3 or more radiologists | 1 | 1.1 (1/90) | 1 | 1.1 (1/90) | 0 | 0 |
| Score 4 or 5 by 3 or more radiologists | 0 | 0 | 0 | 0 | 0 | 0 |
| **Screening examinations with a false positive result in To-Be 1, no cancer in To-Be 2 (n=39)** | **DBT+SM** | | **DBT** | | **SM** | |
| Proportion assigned in a review | n | % (fraction) | n | % (fraction) | n | % (fraction) |
| Score 2 by 1 or more radiologists | 32 | 82.1 (32/39) | 31 | 79.5 (31/39) | 30 | 76.9 (30/39) |
| Score 3 by 1 or more radiologists | 33 | 84.6 (33/39) | 31 | 79.5 (31/39) | 15 | 38.5**(15/39) |
| Score 4 or 5 by 1 or more radiologists | 16 | 41.0 (16/39) | 14 | 35.9 (14/39) | 5 | 12.8 (5/39) |
|  |  |  |  |  |  |  |
| Score 2 by 2 or more radiologists | 27 | 69.2 (27/39) | 17 | 43.6 (17/39) | 22 | 56.4 (22/39) |
| Score 3 by 2 or more radiologists | 17 | 43.6 (17/39) | 14 | 35.9 (14/39) | 8 | 20.5 (8/39) |
| Score 4 or 5 by 2 or more radiologists | 4 | 10.3 (4/39) | 4 | 10.3 (4/39) | 0 | 0 |
|  |  |  |  |  |  |  |
| Score 2 by 3 or more radiologists | 19 | 48.7 (19/39) | 11 | 28.2 (11/39) | 11 | 28.2(11/39) |
| Score 3 by 3 or more radiologists | 6 | 15.4 (6/39) | 6 | 15.4 (6/39) | 3 | 7.7 (3/39) |
| Score 4 or 5 by 3 or more radiologists | 1 | 2.6 (1/39) | 1 | 2.6 (1/39) | 0 | 0 |

^#^ Number shows the number of cases chosen/assigned for each radiologist and different combinations of radiologists, some of these cases are the same, but the cases could also differ for the different radiologists

*p=0.04 for comparison of DBT and SM

**p<0.001 for comparison of DBT and SM

Table C3. Number and proportion of negative screening examinations, and examinations with a false positive result for a score of 2 or higher, and a score of 3 or higher by one or more radiologists, two or more radiologists, and three or more radiologists

|  | **Negative screening examinations (n=90)** | | **Screening examinations with a false positive result (n=39)** | | **Total (n=129)** | |
| --- | --- | --- | --- | --- | --- | --- |
|  | n | % (fraction) | n | % (fraction) | n | % (fraction) |
| Score 2, 3, 4, or 5 by 1, 2, 3, 4, or 5 radiologists | 68 | 75.6 % (68/90) | 38 | 97.4 % (38/39) | 106 | 82.2 % (106/129) |
| **Score 2, 3, 4, or 5 by 2, 3, 4, or 5 radiologists** | **43** | **47.8 % (43/90)** | **35** | **89.7 % (35/39)** | **78** | **60.5 % (78/129)** |
| Score 2, 3, 4, or 5 by 3, 4, or 5 radiologists | 17 | 18.9 % (17/90) | 29 | 74.4 % (29/39) | 46 | 35.7 % (46/129) |
|  |  |  |  |  |  |  |
| Score 3, 4, or 5 by 1, 2, 3, 4, or 5 radiologists | 45 | 50.0 % (45/90) | 35 | 89.7 % (35/39) | 80 | 62.0 % (80/129) |
| Score 3, 4, or 5 by 2, 3, 4, or 5 radiologists | 10 | 11.1 % (10/90) | 24 | 61.5 % (24/39) | 34 | 26.4 % (34/129) |
| Score 3, 4, or 5 by 3, 4, or 5 radiologists | 2 | 2.2 % (2/90) | 11 | 28.2 % (11/39) | 13 | 10.1 % (13/129) |
